# The *MrCYP52* Cytochrome P450 Monoxygenase Gene of *Metarhizium robertsii* Is Important for Utilizing Insect Epicuticular Hydrocarbons

**DOI:** 10.1371/journal.pone.0028984

**Published:** 2011-12-16

**Authors:** Liangcai Lin, Weiguo Fang, Xinggang Liao, Fengqing Wang, Dongzhi Wei, Raymond J. St. Leger

**Affiliations:** 1 State Key Laboratory of Bioreactor Engineering, Newworld Institute of Biotechnology, East China University of Science and Technology, Shanghai, China; 2 Department of Entomology, University of Maryland, College Park, Maryland, United States of America; Universidade de Sao Paulo, Brazil

## Abstract

Fungal pathogens of plants and insects infect their hosts by direct penetration of the cuticle. Plant and insect cuticles are covered by a hydrocarbon-rich waxy outer layer that represents the first barrier against infection. However, the fungal genes that underlie insect waxy layer degradation have received little attention. Here we characterize the single cytochrome P450 monoxygenase family 52 (*MrCYP52*) gene of the insect pathogen *Metarhizium robertsii*, and demonstrate that it encodes an enzyme required for efficient utilization of host hydrocarbons. Expressing a green florescent protein gene under control of the *MrCYP52* promoter confirmed that *MrCYP52* is up regulated on insect cuticle as well as by artificial media containing decane (C10), extracted cuticle hydrocarbons, and to a lesser extent long chain alkanes. Disrupting *MrCYP52* resulted in reduced growth on epicuticular hydrocarbons and delayed developmental processes on insect cuticle, including germination and production of appressoria (infection structures). Extraction of alkanes from cuticle prevented induction of *MrCYP52* and reduced growth. Insect bioassays against caterpillars (*Galleria mellonella*) confirmed that disruption of *MrCYP52* significantly reduces virulence. However, *MrCYP52* was dispensable for normal germination and appressorial formation *in vitro* when the fungus was supplied with nitrogenous nutrients. We conclude therefore that *MrCYP52* mediates degradation of epicuticular hydrocarbons and these are an important nutrient source, but not a source of chemical signals that trigger infection processes.

## Introduction

Although hydrocarbon degrading enzymes are generally associated with oil-polluted environments, alkanes are persistent molecules and this makes them useful components for natural barriers. Thus, both plant and insect cuticles are covered by a waxy epicuticular layer comprising a mixture of long chain alkanes and related chemicals that represent the first barrier to environmental threats [1]. Most plant and insect diseases are caused by fungi that infect their hosts by direct penetration of the cuticle [2], but the fungal genes responsible for waxy layer degradation remain almost completely unexplored. In the rice blast fungus *Magnaporthe oryzae*, a putative alkane degrading cytochrome P450 is up-regulated upon the first stages of infection [3], which implies that alkane degradation is required for breaching host defenses and/or to access nutrients.

The entomopathogens *Metarhizium robertsii* and *Beauveria bassiana* infect a broad array of insects and hence can be exploited as biological control agents of pests. They are able to grow on straight chain and branched hydrocarbons as sole carbon and energy source [4]–[6], and the specific locust pathogen *Metarhizium acridum* extensively hydrolyzes surface lipids during germination and pre-penetration growth on locust cuticles [7]. Pedrini et al [6] postulated that the cytochrome P450 monoxygenases (CYP) are involved in alkane and insect epicuticle degradation, and they showed that *Beauveria bassiana* differentially expressed CYP genes when grown on different hydrocarbons. The versatile CYP52 family (membrane bound class II) contains several enzymes with demonstrated activity towards alkanes and/or fatty acids [1]. Although the genome of *M. robertsii* encodes 123 highly divergent CYP genes [8], *M. robertsii* has only a single CYP52 (MAA_06634) compared to four in *M. acridum* [8].

This study focuses on *M. robertsii* strain ARSEF 2575 (Mr2575). Mr2575 is the type strain of *M. robertsii*, and is frequently employed as a model for studies on host pathogen interactions and genetic engineering. Infection by Mr2575 proceeds via spores that adhere to the host surface (epicuticle) and germinate to form germ tubes that continue undifferentiated hyphal growth if nutrient quality and quantity is not conducive to differentiation. On a host, however, apical elongation terminates and germ tubes produce infection structures, called appressoria, which promote the localized production of cuticle degrading enzymes [9]–[11]. Lipids are the main nutrient reserve in fungal spores [12]. Lipid bodies are transported to the developing appressoria and degraded to release glycerol which increases hydrostatic pressure and provides a driving force for mechanical penetration [11], [13]. Despite their intracellular lipid reserves, Mr2575 spores are nutrient deficient, as they also need extracellular nutrients in order to germinate [9].

In this study we show for the first time that a specific hydrocarbon utilizing enzyme is a virulence factor in an entomopathogenic fungus. The *M. robertsii* CYP52 (*MrCYP52*) increases differentiation of appressoria on grasshopper cuticle, and is required for full pathogenicity, but is expendable for appressorial formation *in vitro* when the fungus is supplied with nitrogenous nutrients. The implication is that epicuticular hydrocarbons are an important nutrient source, but not a source of chemical signals that trigger infection processes.

## Materials and Methods

### Fungal and bacterial strains

*M. robertsii* wild-type strain ARSEF2575 was obtained from the USDA/ARS Collection of Entomopathogenic Fungal Cultures, Ithaca, NY. *Escherichia coli* DH5α was employed for DNA manipulation. *Agrobacterium tumefaciens* AGL-1 was used for *M. robertsii* transformation.

### Gene cloning and disruption

We modified the master Ti vector pFBARGFP used for gene disruption in *M. robertsii* [13], by removing the EcoR I site. The new bar cassette without the lox-NotI-lox site was obtained by PCR using the primers Bar5 and Bar3 (Table S1). The primers were designed to include unique Xba I/EcoR I and Spe I/EcoR V sites at the 5′-end and 3′-end of the bar gene. The resultant fragment was digested with Xba I and EcoR V, and cloned into pFBARGFP to produce pPK2BarGFPD. To construct the vector for *MrCYP52* disruption, the 5′ end and 3′ end of *MrCYP52* were cloned by PCR and inserted into the EcoR I and Spe I sites, respectively, of pPK2BarGFPD. The disruption mutant (*ΔMrCYP52*) was obtained utilizing *Agrobacterium tumefaciens* AGL-1 as described [14]. To complement *ΔMrCYP52*, the genomic sequence of *MrCYP52* including its promoter (2215 bp) and terminator (427 bp) was cloned, inserted into the EcoR V site of pPK2SurGFPD [15], and transformed into *ΔMrCYP52*. Successful disruptant and complementation of strains were confirmed by PCR and Southern blotting (Figure S1). To overexpress of *MrCYP52*, the ORF of the *MrCYP52* gene was amplified by reverse transcription (RT)-PCR with primers MrCYP52_ORF_F and MrCYP52_ORF_R. The *MrCYP52* ORF was digested with Spe I and EcoR I and ligated into pBARGPE1 downstream of the constitutive *gpd* promoter, following which the expression cassette was excised with Not I and Nde I, blunt-end digested with T4 DNA polymerase (New England Biolabs, Pickering, Ontario, Canada), and inserted into the EocR V site of pPK2BarGFPD. The resulting plasmid (pPK2BarGFPD- MrCYP52) was transformed into wild type *M. robertsii*. The primers used in this study were listed in Table S1. All PCR products in this study were cloned into the pGEM-T vector (Promega) and sequenced for confirmation.

### Southern blotting

Southern blotting was performed with 20 µg of DNA for each sample. Genomic DNA was extracted as described previously [16] and digested with EcoR V. Agarose gel electrophoresis and DNA transfers were performed as described [17]. The fragment used as the probe was generated using primers MrCYP52_UR and MrCYP52_CF (Table S1). Probe preparation, membrane hybridization and visualization were according to the manufacturer's instructions (DIG High Prime DNA Labeling and Detection Starter Kit II, Roach).

### Insect bioassay

*M. robertsii* was bioassayed using *Galleria mellonella* larvae from Big Apple Herpetological (Hauppauge, NY) as described [13]. Briefly, insects were inoculated by a 10 second immersion in conidial suspensions (1×10^7^ conidial ml^−1^). All bioassays were repeated three times with 36 insects per replicate. Mortality was recorded every day.

### Utilization of different alkanes

Germination rates of the wild type Mr2575 were compared with *ΔMrCYP52* in basal medium (BS) (0.1% KH_2_PO_4_, 0.025% Na_2_SO_4_, 0.05% KCl, 0.0125% MgSO_4_·7H_2_O, 0.00625% CaCl_2_, and 0.3% NaNO_3_) supplemented with the alkanes listed in Table 1. To obtain insect-derived hydrocarbons, we extracted locust wings using hexane [12], [18]. The extract was dried and the precipitate resuspended in hexane. One hundred microliter hexane extract, the equivalent to 4 locust wings, was evaporated per slide on sterilized glass microscope cavity slides. The slides were inoculated with 40 µl drops of 1×10^6^ spores ml^−1^ and incubated at 27°C. Germination rates were recorded as described above. Mycelial biomasses were determined by inoculating 1.5×10^7^ spores into 50 ml of autoclaved BS medium supplemented with filter sterilized hydrocarbons. Cultures were shaken (200 rpm) at 27°C and harvested by vacuum filtration on pre-weighed glass-fiber filters five days after the first appearance of visible growth. The filters were washed in water and acetone, and dried to constant weight at 80°C [4].

**Table 1:** Germination rate of WT and *ΔMrCYP52* on 1% alkanes and cuticular hydrocarbons (20 hrs).

	Wild type	*ΔMrCYP52*	% reduction in alkane induced growth
BS	10.7±0.8%	10.9±1.1%	
**n-alkanes**			
Nonane (C_9_)	16.7±0.6%	15.8±2.3%	18.3%
Decane (C_10_)	18.9±0.8%	12.9±0.8%	75.6%
Dodecane (C_12_)	33.3±1.5%	32.9±1.8%	2.6%
Tridecane (C_13_)	33.0±1.3%	32.6±1.7%	2.7%
Tetradecane (C_14_)	18.2±1.6%	16.3±1.3%	28%
Pentadecane (C_15_)	23.3±2.9%	19.3±0.3%	33.3%
Hexadecane (C_16_)	31.9±0.6%	25.9±1.3%	28.7%
Hexacosane (C_26_)	39.4±1.0%	32.3±1.4%	25.4%
Octacosane (C_28_)	50.8±2.1%	29.2±2.0%	54.4%
**Cuticular hydrocarbons**			
Hexane extract	57.7±2.1%	35.6±1.3%	47.4%
**Branched alkanes**			
Pristane	24.4±1.1%	23.6±2.6%	7.3%
Squalane	24.9±1.6%	24.2±1.8%	6.3%

### Conidial germination and appressorial differentiation

The germination rate of conidia was measured by inoculating 20 µl of spore suspension (2×10^7^ spores ml^−1^) into 3.5 cm polystyrene petri dishes containing 2 ml of 0.0125% yeast exact (YE). Three hundred spores from each of three replicates were recorded microscopically to assess germination and appressorial differentiation against the hydrophobic surface of the Petri dish. Appressoria were also induced against locust (*Schistocerca gregaria*) hind wings as described previously [12], [19]. Cuticles were inoculated with 3 µl of conidia suspension (5×10^6^ ml^−1^) and incubated on 1.5% water agar.

### RT-PCR analysis

To monitor the expression of *MrCYP52* in different growth conditions, mycelial inoculums from 36 h Sabouraud dextrose broth (SDB) (Difco) cultures [11] were incubated (6 hours) in 50 ml of either water, Sabouraud dextrose broth (SDB), hemolymph or basal medium (BS) containing either 1% glucose, 1% decane or 1% *Manduca sexta* cuticle. The *M. sexta* cuticle and hemolymph were prepared as described previously [12]. RNA was extracted from mycelia using a QIAGEN RNase Plant Mini kit, treated with DNase I, and 1 µg was converted into single-stranded cDNA using Verso™ TR-PCR kit (Thermo Scientific).

### Expression pattern of *MrCYP52*in *M. robertsii*

The expression pattern of *MrCYP52* was investigated in transformants expressing GFP driven by the promoter region (2200 bp) of *MrCYP52*. The promoter region was cloned by PCR using primers MrCYP52_PF and MrCYP52_PR (Table S1), digested with Bgl II and EcoR I and inserted into BamH I/EcoR I sites of pBarGPE1-GFP [20]. The *egfp* cassette was released by Hpa I and Spe I, blunted with T4 DNA polymerase, and inserted into the EcoR V site of pPK2Bar to form pPMrCYP52:GFP. This Ti plasmid was transformed utilizing *A. tumefaciens* into the wild type *M. robertsii* ARSEF2575, producing WT-PMrCYP52: GFP. To study the time course of *MrCYP52* expression, 30 µl 2×10^7^ ml^−1^ spore suspensions of WT-PMrCYP52:GFP were incubated for 10 h in 0.0125% YE and then transferred to BS supplemented with different carbon source (1% liquid alkanes, 1% glucose or 1% glycerol). For solid alkanes, 40 µl 1% long chain alkane or myristic acid solution in hexane was pipette onto glass coverslips and evaporated, leaving a white greasy layer. The coverslips were then placed in polystyrene petri dishes with the alkane layer facing up, and the dishes were inoculated with 30 µl of 1×10^7^ ml^−1^ spore suspensions in BS medium. Spores of WT-PMrCYP52:GFP (5×10^6^ ml^−1^) were also inoculated onto locust wings and to locust wings treated with hexane to remove hydrocarbons. GFP fluorescence was followed microscopically.

## Results

### Protein characteristics and phylogenetic analysis

**Figure 1:**
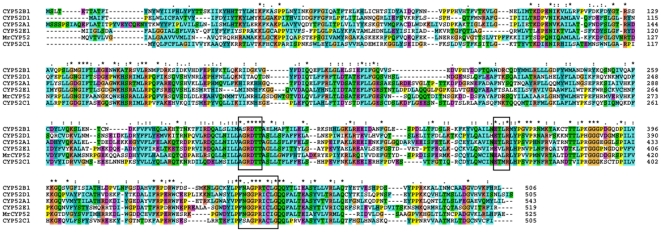
Sequence alignment analysis of *MrCYP52* with defined CYP52s. CYP52A1 (AAA34354), CYP52B1 (CAA78357), CYP52C1 (CAA78358) and CYP52D1 (Q12585) from *Candida tropicalis*, CYP52E1 (P43083) from *Candida apicola*. The heme-binding regions and other conserved domains discussed in the text are marked.

Analyses of the predicted *MrCYP52* protein (EFY97851) [8] indicates that it is composed of 525 amino acid residues (59.5 kDa) with a predicted pI of 8.57. According to TMpred analysis (Figure S2), the predicted protein resembles other fungal cytochrome P450s in having a single hydrophobic transmembrane domain at the N-terminus, and it contains the signature pfam00067 Cytochrome P450 conserved domain (Figure 1). A neighbor-joining analysis of 28 fungal CYPs conducted with MEGA4 (Figure S3) showed that *MrCYP52* clusters within the alkane hydroxylating CYP52 family, and is similar to CYP52 enzymes from *Beauveria bassiana* (ADK36660, 59% identity), *Candida maltose* (AAA34320, 44% identity), and *Candida tropicalis* (XP_002546279, 45% identity). Homologs (>40% identity) of *MrCYP52* were identified in most other Ascomycete fungi, but were absent in the genomes of Basidiomycete, Chytrid and Zygomycete fungi, showing that the gene is expendable in some lifestyles.

### Alkane utilization assay

To study the function of *MrCYP52*, *MrCYP52* null mutants (*ΔMrCYP52*) were generated in Mr2575 by homologous replacement. Complementing *ΔMrCYP52* with a genomic clone of *MrCYP52* produced a strain indistinguishable from the wild type in all the phenotypic assays performed in this study. Consequently, the data for the complemented strains is not presented.

**Figure 2:**
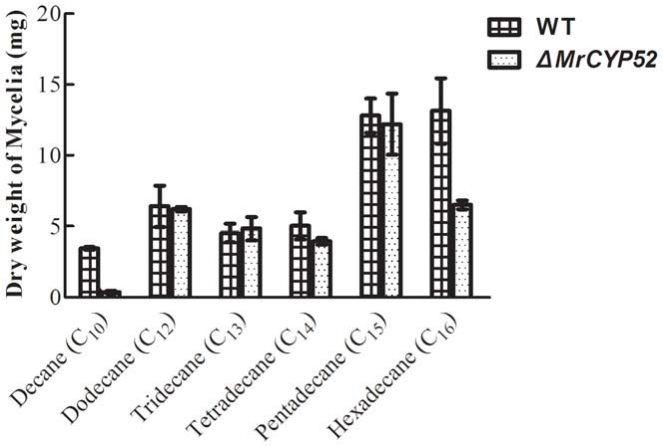
Growth of *M. robertsii* wild type and *ΔMrCYP52* in basal salts containing 1% alkanes.

To investigate the role of *MrCYP52* in utilizing alkanes, we compared the germination rate of the wild type with that of *ΔMrCYP52* in BS medium (containing no carbon source) supplemented with each of the alkanes listed in Table 1. Linear alkanes, C9, C12–C15, and branched alkanes increased germination of wild type and *ΔMrCYP52* spores to a similar extent. In contrast, C10 did not significantly increase germination of *ΔMrCYP52* above the very low levels seen in BS medium (*P>0.05*). Germination rates of *ΔMrCYP52* on C16, C26, C28 and locust cuticle hexane extract were significantly reduced compared with the wild type (*P<0.05*). *ΔMrCYP52* also grew poorly in liquid cultures containing C10 and C16 (Figure 2), indicating that *MrCYP52* participates in mid and long chain alkane utilization.

### Germination behavior and appressorial formation

Pathogenicity assays involving topical application of spores (the normal route of entry) to *Galleria mellonella* caterpillars revealed that *ΔMrCYP52* (LT_50_ = 8.27±0.32 d) took a significantly (*P<0.05*) longer time to kill than the wild type *M. robertsii* 2575 (LT_50_ = 6.85±0.37 d). Although alkane-growth adaptation of *B. bassiana* is correlated with increased insect host mortality [21], overproduction of the *MrCYP52* gene did not increase the pathogenicity of *M. robertsii* (LT_50_ = 6.92±0.18 d). To elucidate whether *MrCYP52* is needed for pre-penetration developmental processes, wild type Mr2575 and *ΔMrCYP52* strains were grown in 0.0125% yeast extract (YE) which induces differentiation of appressoria by WT Mr2575 [9]. There was no significant difference in sporulation, germination, growth rates and differentiation of appressoria between *M. robertsii* 2575 and *ΔMrCYP52*, indicating that *MrCYP52* does not facilitate these processes in the presence of low levels of nitrogenous nutrients (Table S2). However, the germination rate of *ΔMrCYP52* on locust cuticle was significantly (*P<0.001*) slower than that of wild type *M. robertsii* (Table S2), and 6 h post inoculation only 24.6±0.7% of *ΔMrCYP52* conidia had germinated as compared to 40.4±0.3% of the wild type Mr2575. Both wild type and *ΔMrCYP52* had 100% germination rates 12 h post inoculation. However, 25 hours post inoculation, the wild type Mr2575 had produced ∼2.2-fold more appressoria than *ΔMrCYP52*. By three days post-inoculation differences were still significant, with 43.5±1.4% of *ΔMrCYP52* germlings having produced appressoria as compared to 52.9±1.5% of the wild type. The germination rate of *ΔMrCYP52* and the wild type were similarly low on the locust wings treated with hexane to remove cuticular hydrocarbons (Table S2). To determine if addition of exogenous nutrients overcomes the reduced ability of *ΔMrCYP52* to infect locust cuticle, we inoculated cuticles with *ΔMrCYP52* spores suspended in 0.0125% YE. Exogenous nutrients increased the germination rate of wild type (from 40.4±0.3 to 57.6±1.2%) and *ΔMrCYP52* (from 24.6±0.7 to 54.1±2.3%). Interestingly, YE increased differentiation of infection structures by *ΔMrCYP52* to the levels shown by the wild type Mr2575 in the absence of YE, but appressorial formation by the wild type was significantly (*P<0.05*), reduced (by 37.5±1.2%) by additional nutrition.

### Expression of *MrCYP52*

**Figure 3:**
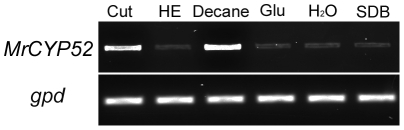
Expression of *MrCYP52*. RT-PCR analysis of *MrCYP52* expression by Wild type *M. robertsii* transferred from SDB medium to water, SDB, cell free hemolymph (HE), basal medium (BS) containing 1% *Manduca* larval cuticle (Cut), 1% decane or 1% glucose (MM) for 6 h.

**Figure 4:**
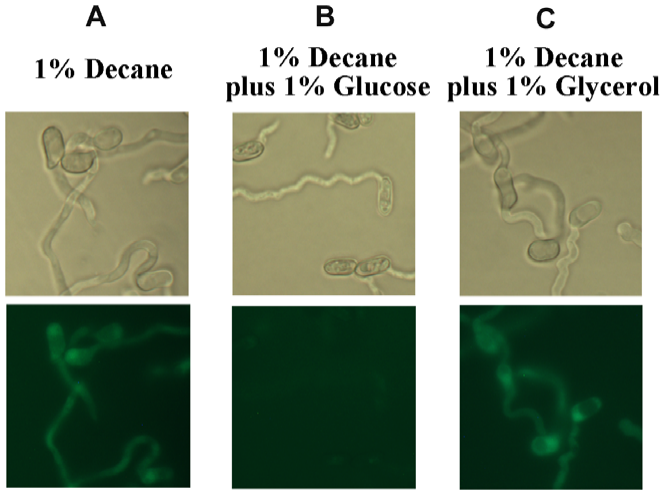
Induction of WT-PMrCYP52:GFP incubated for one hour. (A) 1% decane. (B) 1% decane supplemented with 1% glucose. (C) 1% decane supplemented 1% glycerol.

**Figure 5:**
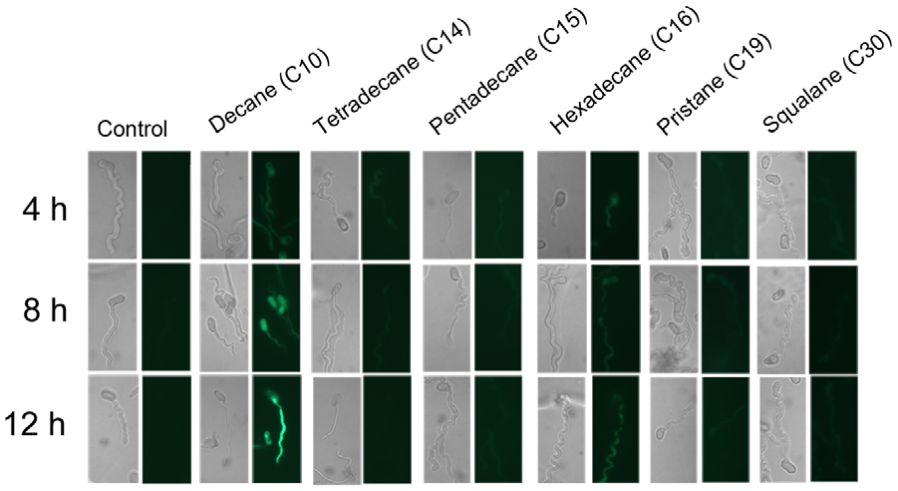
Time course of induction of WT-PMrCYP52:GFP in BS supplemented with different alkanes. 30 µl 2×10^7^ ml^−1^ spore suspensions of WT-PMrCYP52:GFP were incubated for 10 h in 0.0125% YE and then transferred to BS supplemented with different carbon source.

**Figure 6:**
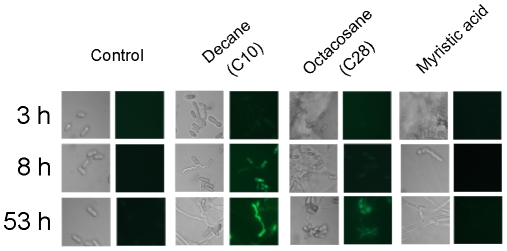
Time course of induction of WT-PMrCYP52:GFP in BS supplemented with decane, octacosane or myristic acid. 40 µl 1% octacosane or myristic acidsolution in hexane was pipette onto glass coverslips and evaporated, leaving a white greasy layer. The coverslips were then placed in polystyrene petri dishes with the alkane layer facing up, and the dishes were inoculated with 30 µl of 1×10^7^ ml^−1^ spore suspensions in BS medium.

**Figure 7:**
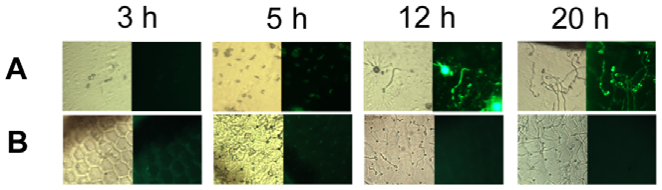
Induction of WT-PMrCYP52:GFP on locust wings (A) or locust wings treated with hexane(B).

RT-PCR analyses demonstrated that *MrCYP52* was expressed in diverse nutrient rich and nutrient poor media (Figure 3). The time course for *MrCYP52* expression was also examined by following GFP fluorescence driven by the *MrCYP52* promoter in WT-PMrCYP52:GFP. GFP fluorescence was not observed in BS medium without a carbon source. Spores of WT-PMrCYP52:GFP pre-germinated in 0.01% YE fluoresced within 1 h of transfer to BS containing 1% decane or 1% decane plus 1% glycerol. In contrast, GFP fluorescence was not observed in BS containing 1% decane plus 1% glucose (Figure 4), indicating that expression is repressed by glucose. Faint GFP fluorescence was observed within 4 hours when medium-chain alkanes (n-alkanes or branched alkanes) were used as sole carbon source (Figure 5). Spores inoculated directly into BS medium containing C28 as carbon source fluoresced faintly after 8 hours (Figure 6). However, GFP fluorescence could not be observed in BS medium with myristic acid as carbon source (Figure 6), which suggests that *MrCYP52* may not have an important role in metabolizing fatty acids. WT-PMrCYP52:GFP germinated at the same rate as the wild type when inoculated onto locust cuticle. GFP fluorescence could be observed in conidia germinating 5 hours post inoculation, and the fluorescence increased in intensity as germlings grew and differentiated appressoria. In contrast, fluorescence could not be observed when WT-PMrCYP52:GFP was inoculated onto locust wings treated with hexane (Figure 7).

## Discussion

The epicuticle (outer insect waxy layer) is the interface between the insect and its environment, and the first barrier an entomopathogenic fungi needs to cross in order to infect an insect. Hydrocarbons, mostly alkanes in the chain length range C21–C35, comprise over 90% of the epicuticular layer on the surface of grasshoppers, with the balance being composed of wax esters, free fatty acids and triacylglycerides [22]–[23]. Although several aspects of the interactions between entomopathogenic fungi and insect host epicuticular hydrocarbons have been examined, the fungal genes that underlie insect waxy layer degradation remain almost completely unexplored [6]. Cytochrome P450s constitute a superfamily of monoxygenases involved in the hydroxylation of a wide range of endogenous and xenobiotic compounds, including fatty acids and alkanes (metabolism of alkanes is coupled to fatty acid degradation as conversion of alkanes to fatty acids is an essential step in the alkane assimilation process). Besides their involvement in different physiological processes, P450s also differ in the position of the hydroxylation; this can happen close to the carboxyl group (mediated by CYP152), can occur in-chain (e.g., CYP1006) or at the terminal or subterminal end (e.g., CYP52). Blast searches of *M. robertsii* genome revealed that among its 123 CYP enzymes [8] there was a single CYP1006 with similarity (46% identity) to a diol synthase (CYP1006C1) from *Aspergillus nidulans*, and one hydroxylase belonging to the CYP52 family (membrane bound class II). There were no CYP152s. Several CYP52 enzymes have activity towards alkanes and/or fatty acids [1]. However, no induction of *MrCYP52* was observed for mytistic acid, suggesting that its main function is not omega-hydroxylation of fatty acids. Alkanes are predominantly oxidized by microbes at a terminal methyl group, which represents the first and rate-limiting step in the alkane degradation pathway [1]. The importance of monoterminal oxidation to *M. robertsii* was shown by its ability to utilize 2-methyl but not 3-methylnonane, due to the alkyl branch at the β position causing steric inhibition of terminal oxidizing enzymes [4]. The involvement of CYP52s in terminal hydroxylation of n-alkanes implicated *MrCYP52* as a potential contributor to the degradation of epicuticular lipids. Compared to *ΔMrCYP52*, wild type Mr2575 germinated faster with a cuticle-derived hydrocarbon extract or long chain alkanes, while expression of *MrCYP52* in the wild type also boosted germination as well as appressorial differentiation on locust cuticle. These findings indicate that *MrCYP52* is required by *M. robertsii* for rapid hydrolysis of alkanes and that this provides nutrition for germination and formation of infection structures. The expression of *MrCYP52* shares common features with alkane-degrading P450 genes of *Candida maltose*, such as induction by alkanes and repression by glucose [24], and is consistent with alkanes playing an important role in nutrition in the absence of more readily utilized nutrients. The nutrient poor conditions that allow expression of *MrCYP52* are a necessary trigger for virulence as *M. robertsii* strain Mr2575 only produces infection structures on cuticle surfaces with low levels of nutrients [9]. We identified three putative CREA-binding sites upstream of the *MrCYP52* translation start codon (Figure S4). A trans-acting DNA-binding protein *CRR1* with significant sequence similarity of *A. nidulans CREA* has been demonstrated to effect carbon metabolite repression in *M. robertsii* [25].

Utilizing an *MrCYP52* promoter-reporter construct to precisely reveal the spatial and temporal pattern of *MrCYP52* activity enabled us to confirm that *MrCYP52* is highly expressed throughout the formation of infection structures, suggesting constant induction. Nevertheless, *in vitro*, long chain alkanes were comparatively poor inducers of *MrCYP52*, as compared to decane which is not reported to be a component of the epicuticle. *ΔMrCYP52* did not grow on decane suggesting that *MrCYP52* is required for its assimilation, whereas growth of *ΔMrCYP52* on longer chain alkanes was reduced but not eliminated suggesting that multiple enzymes are involved in the assimilation of molecules longer than decane. *CYP52* enzymes are often length specific [26], so it is possible that fungi have evolved a multitude of currently unknown enzymes that catalyze oxygenation of long chain alkanes to produce decane, and the induced *MrCYP52* has a principal role in providing nutrition by oxygenation of short-chain breakdown products. Consistent with this interpretation, *B. bassiana* produces large amount of decane when grown on the insect-derived hydrocarbon n-octacosane [27]. *MrCYP52* is able to catalyze the degradation of sufficient cuticular hydrocarbons to enhance growth, as shown by the comparatively poor performance of *ΔMrCYP52*. Cuticle treated with hexane to remove hydrocarbons did not induce *MrCYP52* and reduced growth of the wild type fungus to the same level as *ΔMrCYP52*, while *ΔMrCYP52* grew poorly on the extracted hydrocarbons as compared to the wild type, confirming the link between cuticular hydrocarbons, induction of *MrCYP52* and growth. *MrCYP52* homologs were found in many Ascomycete fungi, including species of *Beauveria*, *Magnaporthe*, *Fusarium*, *Aspergillus*, *Glomerella* (*Colletotrichum*) and *Penicillium* that are insect and plant pathogens or soil-dwelling saprophytes, suggesting that *MrCYP52* homologs may be generally important for utilizing alkane components of the epicuticular barriers surrounding plants and insects. Homologs were absent in the plant symbionts *Epichloe* and *Trichoderma*, as well as *Neurospora*, which preferentially colonizes scorched dead plant material [28], showing that the gene is expendable for some Ascomycete life-styles.

## Supporting Information

Figure S1 **Disruption of** ***MrCYP52*** **in** ***M. robertsii*** **using the Bar gene.** (A) Strategy for targeted disruption of *MrCYP52*. Arrows indicate location of PCR primers. (B) Confirmation of the disruption of *MrCYP52* by Southern blot analysis. 1: the wild type strain; 2: the *MrCYP52* disruptant strain; 3: *ΔMrCYP52* was complemented with the wild type *MrCYP52*; (C) Confirmation of the disruption of *MrCYP52* by PCR analysis. M: the *MrCYP52* disruptant strain; W: the wild type strain. MrCYP52_CUP+BarDCUP and MrCYP52_CUP+MrCYP52_CR indicate primer pairs used for PCR amplification.
(TIFF)

Figure S2 **TMpred hydrophilicity analysis of** ***MrCYP52.*** **Shown is a plot of the** *MrCYP52* **amino acid numbers (X coordinate) against the probability that a specific amino acid is part (positive values on the y coordinate) or not part (negative values on the y coordinate) of a transmembrane helix. Solid and dotted lines indicated the probability of a putative transmembrane helix to be oriented from the cytoplasm toward the outside (i→o) or in the opposite direction (o→i), respectively.**
(TIFF)

Figure S3 **Phylogenetic analysis of** ***MrCYP52*** **along with known fungal cytochrome P450 encoding genes.** MEGA4 software was used to carry out the analysis. Bootstrap values are adjacent to each internal node, representing the percentage of 1,000 bootstrap replicates.
(TIFF)

Figure S4 **Nucleotide sequence of the 5′-upstream region of the** ***MrCYP52*** **gene.** The translation start point is shown in bold. The putative CREA-binding sites are underlined.
(TIFF)

Table S1 **Primers used in this study.**
(DOCX)

Table S2 **Germination and appressorial formation (in brackets) against plastic or insect cuticle.**
(DOCX)
