# Supplementary figures and images for: The MrCYP52 Cytochrome P450 Monoxygenase Gene of Metarhizium robertsii Is Important for Utilizing Insect Epicuticular Hydrocarbons
Source: PLoS One. 2011 Dec 16;6(12):e28984. doi: 10.1371/journal.pone.0028984 (PMC3241696; doi:10.1371/journal.pone.0028984)

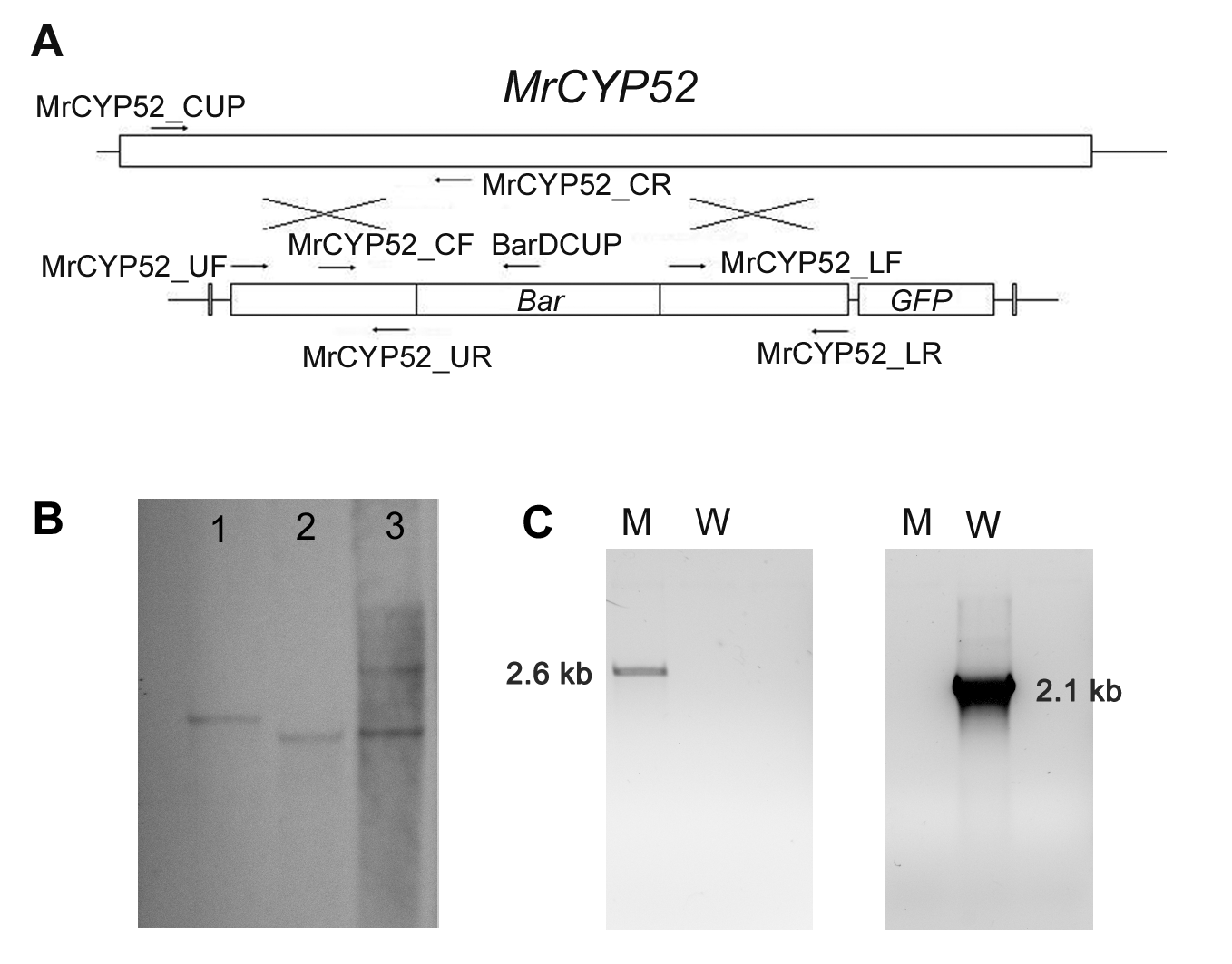

Supplement: Figure S1 — Disruption of MrCYP52 in M. robertsii using the Bar gene. (A) Strategy for targeted disruption of MrCYP52. Arrows indicate location of PCR primers. (B) Confirmation of the disruption of MrCYP52 by Southern blot analysis. 1: the wild type strain; 2: the MrCYP52 disruptant strain; 3: ΔMrCYP52 was complemented with the wild type MrCYP52; (C) Confirmation of the disruption of MrCYP52 by PCR analysis. M: the MrCYP52 disruptant strain; W: the wild type strain. MrCYP52_CUP+BarDCUP and MrCYP52_CUP+MrCYP52_CR indicate primer pairs used for PCR amplification. (TIFF) [file pone.0028984.s001.tiff]

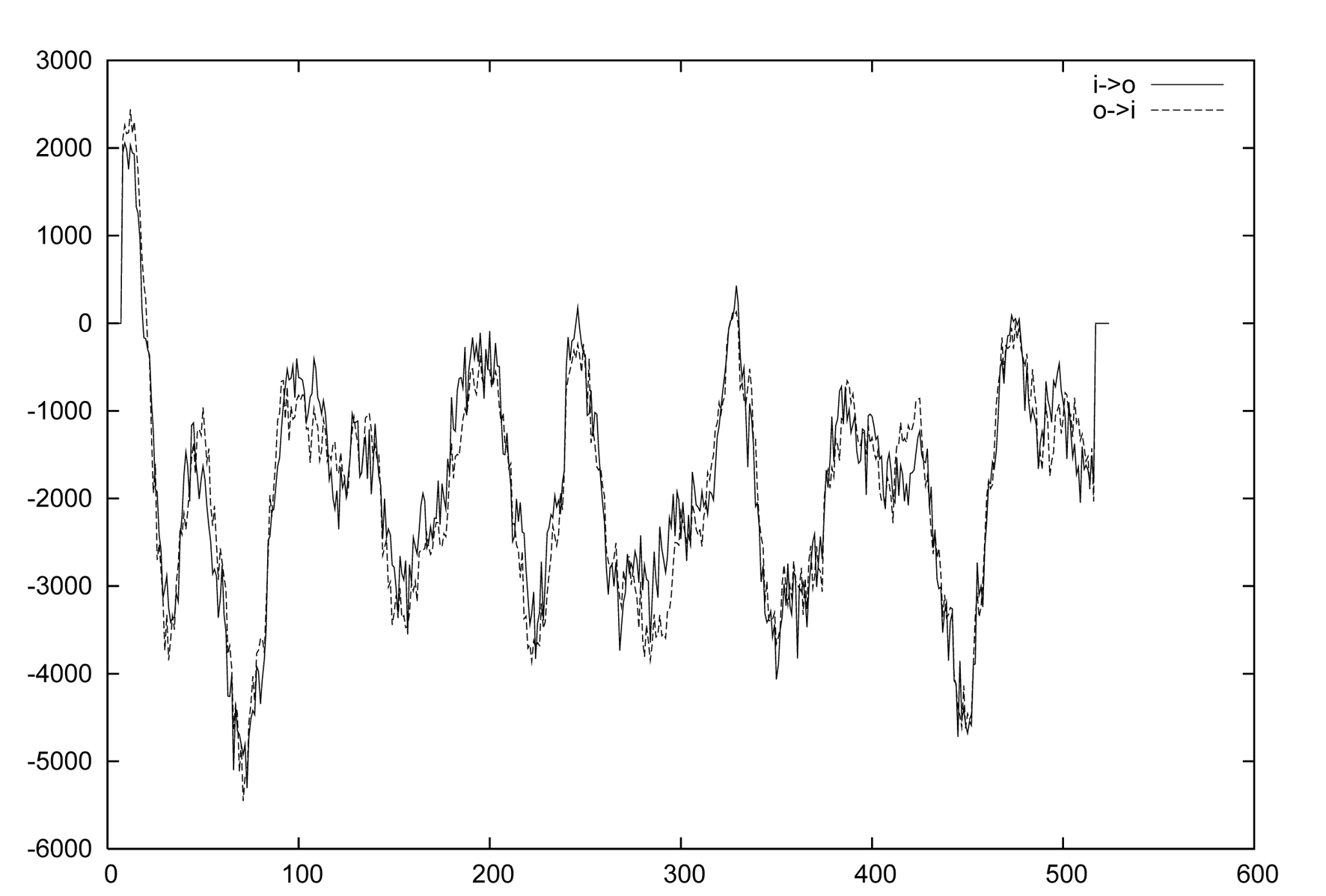

Supplement: Figure S2 — TMpred hydrophilicity analysis of MrCYP52. Shown is a plot of the MrCYP52 amino acid numbers (X coordinate) against the probability that a specific amino acid is part (positive values on the y coordinate) or not part (negative values on the y coordinate) of a transmembrane helix. Solid and dotted lines indicated the probability of a putative transmembrane helix to be oriented from the cytoplasm toward the outside (i→o) or in the opposite direction (o→i), respectively. (TIFF) [file pone.0028984.s002.tiff]

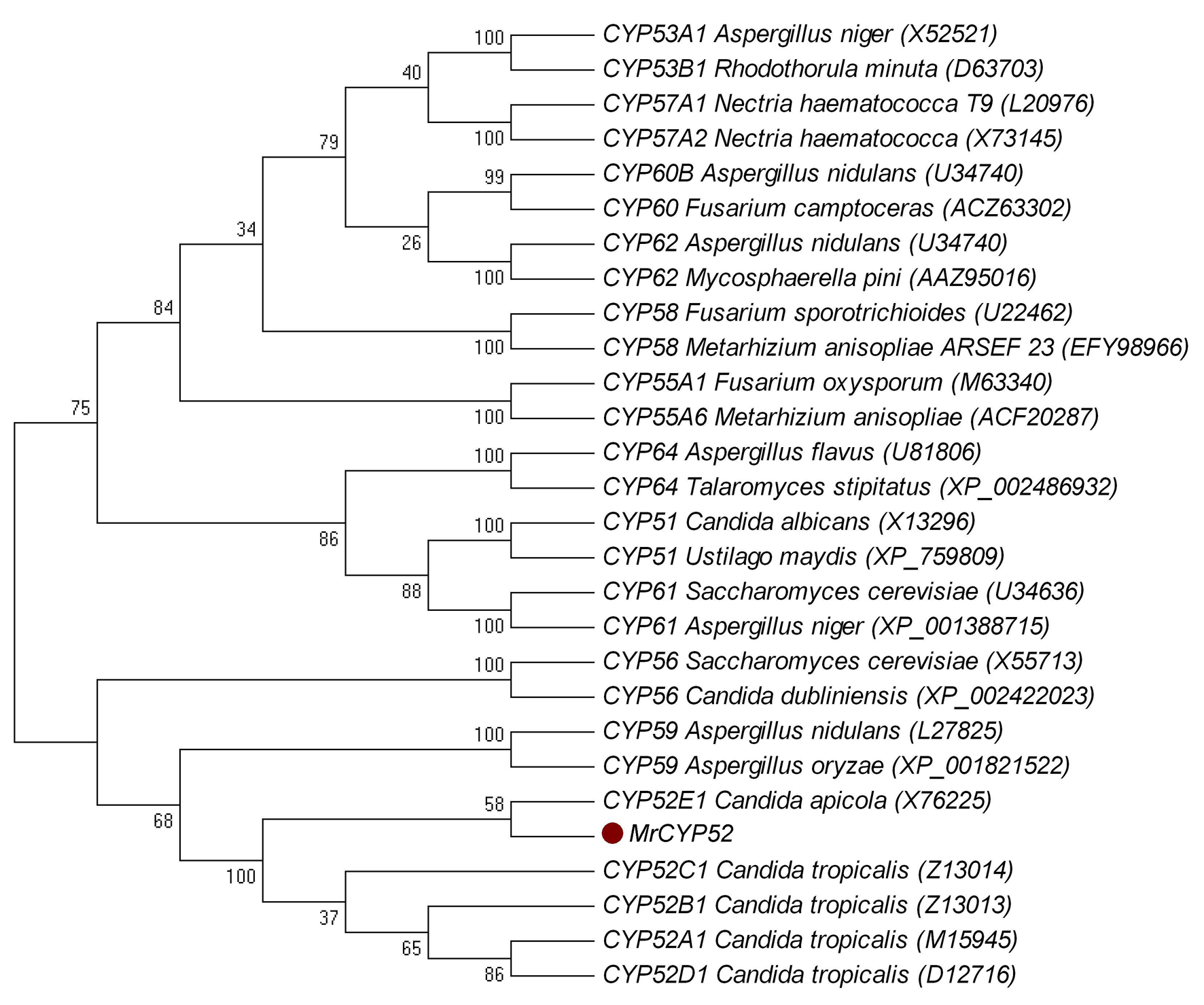

Supplement: Figure S3 — Phylogenetic analysis of MrCYP52 along with known fungal cytochrome P450 encoding genes. MEGA4 software was used to carry out the analysis. Bootstrap values are adjacent to each internal node, representing the percentage of 1,000 bootstrap replicates. (TIFF) [file pone.0028984.s003.tiff]

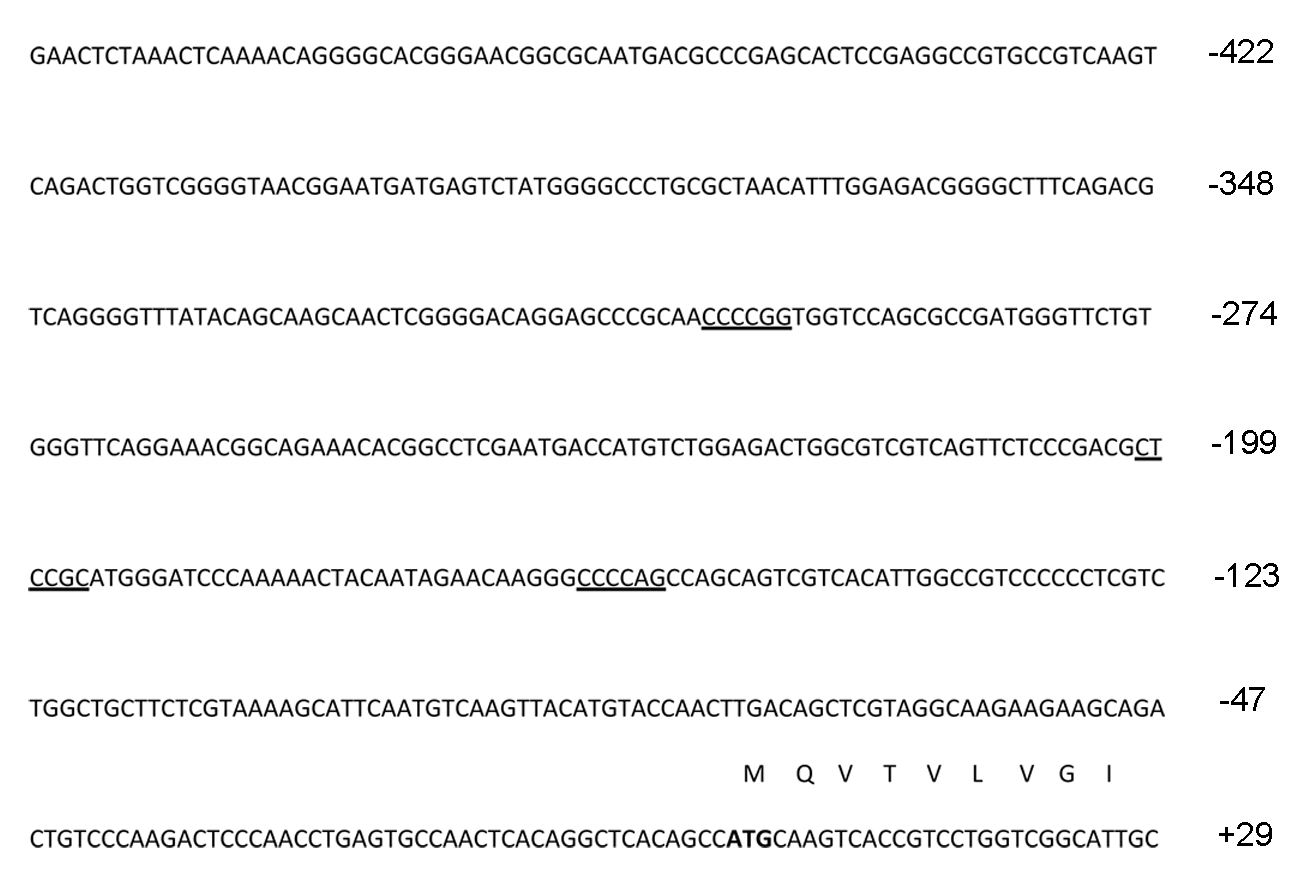

Supplement: Figure S4 — Nucleotide sequence of the 5′-upstream region of the MrCYP52 gene. The translation start point is shown in bold. The putative CREA-binding sites are underlined. (TIFF) [file pone.0028984.s004.tiff]
