# Supplementary material for: The MrCYP52 Cytochrome P450 Monoxygenase Gene of Metarhizium robertsii Is Important for Utilizing Insect Epicuticular Hydrocarbons
Source: PLoS One. 2011 Dec 16;6(12):e28984. doi: 10.1371/journal.pone.0028984 (PMC3241696; doi:10.1371/journal.pone.0028984)
Supplement: Table S1 — Primers used in this study. (DOCX) [file pone.0028984.s005.docx]

**Table S1.** Primers used in this study.

| Name | Sequence | Usage | Remark |
| --- | --- | --- | --- |
| MrCYP52_UF | GCATCATGCATCAGGACATC | The disruption of *MrCYP52* |  |
| MrCYP52_UR | GGCTGTGAGCCTGTGAGTTG | The disruption of *MrCYP52* |  |
| MrCYP52_LF | CACCCTGACCAACTGCCC | The disruption of *MrCYP52* |  |
| MrCYP52_LR | GAAGCCACTGGCTGGTGT | The disruption of *MrCYP52* |  |
| MrCYP52_CUP | TCGGGTTCACCACTTTATTC | Confirm the disruption of *MrCYP52* |  |
| MrCYP52_CF | CGGGCGACGACAGCAACATT | Confirm the disruption of *MrCYP52* |  |
| MrCYP52_CR | CGAGGAGCGGTCTGAAGTTG | Confirm the disruption of *MrCYP52* |  |
| BarDCUP | CAAGTGGGGCTGATCTGACCAG | Confirm the disruption of *MrCYP52* |  |
| MrCYP52_PF | agatctCTCCGCCAGCTCCAGTT | Cloning the promoter of *MrCYP52* to drive gfp and Cloning *MrCYP52* and its promoter and terminator to complement *ΔMrCYP52* | Bgl II |
| MrCYP52_PR | gaattcGGCTGTGAGCCTGTGAGTT | Cloning the promoter of *MrCYP52* to drive gfp | EcoR I |
| MrCYP52_ComR | actagtGCGTCTCTCGGTCTACAGC | Cloning *MrCYP52* and its promoter and terminator to complement *ΔMrCYP52* | Spe I |
| MrCYP52_ORF_F | actagtATGCAAGTCACCGTCCTGGT | Cloning the ORF of *MrCYP52* and RT-PCR | Spe I |
| MrCYP52_ORF_R | gaattcCTACTCGGCGAAATGGAGGC | Cloning the ORF of *MrCYP52* and RT-PCR | EcoR I |
| gpdF | GACTGCCCGCATTGAGAAG | RT-PCR |  |
| gpdR | AGATGGAGGAGTTGGTGTTG | RT-PCR |  |
| BAR5 | tctagagatcgaattcCTCGACAGAAGATGATATTG | Construction Ti vector pPK2BarGFPD | Xba I and EcoR I |
| BAR3 | gatatcgatcactagtACAGGATTCAATCTTAAG | Construction Ti vector pPK2BarGFPD | EcoR V and Spe I |
