# Supplementary material for: The MrCYP52 Cytochrome P450 Monoxygenase Gene of Metarhizium robertsii Is Important for Utilizing Insect Epicuticular Hydrocarbons
Source: PLoS One. 2011 Dec 16;6(12):e28984. doi: 10.1371/journal.pone.0028984 (PMC3241696; doi:10.1371/journal.pone.0028984)
Supplement: Table S2 — Germination and appressorial formation (in brackets) against plastic or insect cuticle. (DOCX) [file pone.0028984.s006.docx]

**Table S2.** Germination and appressorial formation (in brackets) against plastic or insect cuticle.

|  |  | Insect cuticle | | | | | | | | | | |  | Plastic surface | | |
| --- | --- | --- | --- | --- | --- | --- | --- | --- | --- | --- | --- | --- | --- | --- | --- | --- |
|  |  | Insect cuticle | | |  | +0.0125%YE | | |  | -Hydrocarbons (cuticle extracted with hexane) | | |  |  |  |  |
| Time (h) |  | WT (%) |  | *ΔMrCYP52* (%) |  | WT (%) |  | *ΔMrCYP52* (%) |  | WT (%) |  | *ΔMrCYP52* (%) |  | WT (%) |  | *ΔMrCYP52* (%) |
| 3 |  | 0 (0) |  | 0 (0) |  | 0 (0) |  | 0 (0) |  | 0 (0) |  | 0 (0) |  | 0 (0) |  | 0 (0) |
| 6 |  | 40.4±0.3 (0) |  | 24.6±0.7 (0) |  | 57.6±1.2 (0) |  | 54.1±2.3 (0) |  | 21.3±0.9 (0) |  | 23.5±0.7 (0) |  | 37.7±1.4 (0) |  | 38.4± 1.0 (0) |
| 12 |  | 100 (0) |  | 100 (0) |  | 100 (0) |  | 100 (0) |  | 62.5±0.7 (0) |  | 61.4±0.5 (0) |  | 100 (0) |  | 100 (0) |
| 25 |  | 100 (26.0±0.5) |  | 100 (11.5±0.6) |  | 100 (11.1±1.2) |  | 100 (22.9±1.2) |  | 100 (0) |  | 100 (0) |  | 100 (89.8±1.4) |  | 100 (88.9±1.8) |
| 50 |  | 100 (40.8±1.5) |  | 100 (28.9±1.1) |  | 100 (15.2±0.5) |  | 100 (37.2±1.3) |  | 100 (17.7±1.0) |  | 100 (18.3±1.3) |  | 100 (96.5±1.5) |  | 100 (97.6±0.9) |
| 73 |  | 100 (52.9±1.5) |  | 100 (43.5±1.4) |  | 100 (37.5±1.2) |  | 100 (52.5±1.5) |  | 100 (26.8±1.5) |  | 100 (25.8±1.4) |  |  |  |  |
